# ER Dysfunction and Protein Folding Stress in ALS

**DOI:** 10.1155/2013/674751

**Published:** 2013-11-13

**Authors:** Soledad Matus, Vicente Valenzuela, Danilo B. Medinas, Claudio Hetz

**Affiliations:** ^1^Neurounion Biomedical Foundation, Santiago, Chile; ^2^Biomedical Neuroscience Institute, Faculty of Medicine, University of Chile, Santiago, Chile; ^3^Center for Molecular Studies of the Cell, Program of Cellular and Molecular Biology, Institute of Biomedical Sciences, University of Chile, Santiago, Chile; ^4^Department of Immunology and Infectious Diseases, Harvard School of Public Health, Boston, MA, USA

## Abstract

Amyotrophic lateral sclerosis (ALS) is the most frequent paralytic disease in adults. Most ALS cases are considered sporadic with no clear genetic component. The disruption of protein homeostasis due to chronic stress responses at the endoplasmic reticulum (ER) and the accumulation of abnormal protein inclusions are extensively described in ALS mouse models and patient-derived tissue. Recent studies using pharmacological and genetic manipulation of the unfolded protein response (UPR), an adaptive reaction against ER stress, have demonstrated a complex involvement of the pathway in experimental models of ALS. In addition, quantitative changes in ER stress-responsive chaperones in body fluids have been proposed as possible biomarkers to monitor the disease progression. Here we review most recent advances attributing a causal role of ER stress in ALS.

## 1. Introduction

Several neurodegenerative disorders, including Alzheimer's disease, Parkinson's disease, Huntington's disease, and amyotrophic lateral sclerosis (ALS), share common features, among them the presence of abnormal protein aggregates and the inclusions containing specific misfolded proteins. The presence of these abnormal protein aggregates has been temporally and spatially correlated with the activation of stress signaling pathway emerging from the endoplasmic reticulum (ER), a cellular reaction named the “unfolded protein response” (UPR). In the last years, ER stress levels and UPR activation in neurodegenerative diseases have been extensively studied. In this review, we focus on recent findings placing ER stress as a key component of neurodegeneration in ALS and discuss the different mechanisms by which the UPR may impact disease progression and the therapeutic potential of manipulating this signaling pathway in ALS.

## 2. Amyotrophic Lateral Sclerosis

ALS is a progressive and deadly adult-onset motoneuron disease characterized by muscle weakness, spasticity, atrophy, paralysis, and premature death [1, 2]. The pathological hallmark of ALS is the selective degeneration of motoneurons in the spinal ventral horn, most of brainstem nuclei, and cerebral cortex. ALS has an average age of onset around 50 years and estimated incidence of 1-2 cases per 100,000 individuals [1]. ALS is presently incurable with a mean survival time of 1–5 years from diagnosis, often resulting in fatal respiratory dysfunction. The majority of ALS patients lack a defined hereditary genetic component and are considered sporadic (sALS), while approximately 10% of cases are familial (fALS) [1]. The most common genetic causes of fALS are the recently defined hexanucleotide repeat expansion in the intronic region of *C9orf72* and the mutations in the gene encoding cytosolic superoxide dismutase 1 (*SOD1*), which together account for around 50% of fALS cases. Many other disease-causative genes have been identified, including TAR DNA-binding protein (TARDBP or TDP-43), fused in sarcoma (FUS/TLS), vesicle-associated membrane protein-associated protein B (VAPB), among others [1, 3]. All of these mutations trigger the aggregation of the affected protein, which is associated in part with a gain of neurotoxic activity and possibly neuroinflammatory processes. Overexpression of human fALS-linked SOD1 and TDP-43 mutants in mice recapitulates essential features of the human pathology, provoking age-dependent protein aggregation, paralysis, motoneuron degeneration, and muscle atrophy (reviewed in [2, 4]). Studies in these mouse models of ALS have revealed valuable information about the molecular bases of the disease and, in particular, how the presence of these mutant proteins can trigger ER stress.

Since the same groups of neurons are affected in sALS and fALS leading to a similar pathology, it is predicted that therapies in mutant ALS genetic models may translate to sporadic ALS. In fact, accumulation of misfolded oligomers or protein inclusions containing wild-type (WT) TDP-43, FUS, or SOD1 has been recently shown to be a prominent histopathological feature of sALS (see examples in [5, 6]). Different pathogenic mechanisms have been proposed in ALS including neuroinflammation, glial activation, neuronal trafficking problems, excitotoxicity, mitochondrial dysfunction, and oxidative stress (reviewed in [2, 4]). Interestingly, accumulating evidence from several laboratories points towards a key role of alterations of protein homeostasis in the disease process, in both sALS and fALS (reviewed in [7–9]). In this context, ER stress is emerging as an interesting target for the development of prototypic treatments to ALS. In the next sections, we provide a comprehensive update of the work implicating ER stress to ALS pathogenesis.

## 3. ER Stress and UPR Signaling: An Overview

The ER is the first compartment where secreted and membrane proteins are synthesized and folded. For this process, a large and efficient network of chaperones, foldases, and co-factors are expressed at the ER to promote folding and prevent abnormal aggregation of proteins. The ER also operates as a major intracellular calcium store and plays a crucial role in the synthesis of lipids. A number of stress conditions can interfere with the function of this organelle and cause abnormal oxidative folding at the ER lumen, resulting in a cellular condition termed “ER stress” [10]. ER stress engages the unfolded protein response (UPR), an integrated signal transduction pathway that reestablish homeostasis by increasing the protein folding capacity and quality control mechanisms of the ER [11]. Conversely, chronic ER stress results in apoptosis of irreversibly damaged cells through diverse complementary mechanisms [12]. 

The UPR is activated by three main stress sensors, including PKR-like ER kinase (PERK), inositol-requiring transmembrane kinase/endonuclease (IRE1), and activating transcription factor 6 (ATF6). IRE1 is an ER located kinase and endoribonuclease conserved from yeast to humans. Upon UPR activation, IRE1 initiates the splicing of the mRNA encoding the transcriptional factor X-Box-binding protein 1 (XBP1), converting it into a potent activator of multiple UPR-responsive genes (termed XBP1s) [13–15]. XBP1s control the expression of genes involved in protein folding, secretion, protein quality control, and ER-associated degradation (ERAD) [16, 17]. IRE1*α* also regulates other signaling events including the downstream activation of JNK, modulating apoptosis and autophagy levels. In addition, IRE1 is able to degrade a subset of mRNA through its RNAse activity on a tissue specific manner (reviewed in [18]). 

The activation of the stress sensor PERK reduces protein translation into the ER by phosphorylating eukaryotic initiation factor 2 alpha (eIF2*α*), which in turns contributes to decrease the misfolded protein overload [19]. The phosphorylation of eIF2*α* also allows the expression of activating transcription factor 4 (ATF4), a key factor that upregulates a subset of UPR-targeted genes involved in amino acid and redox metabolism, autophagy, protein folding, and apoptosis [20–22] (reviewed in [11, 23]). Among them, CHOP is a key mediator of apoptosis under ER stress [11, 23], which may operate by controlling the expression of several pro-apoptotic members of the BCL2 family of proteins (i.e., BIM and PUMA) in addition to GADD45 [24]. Sustained PERK signaling also contributes to apoptosis by enhancing oxidative stress and by resuming protein synthesis after prolonged ER stress [25–27]. 

ATF6 is activated at the ER and then translocates to the Golgi apparatus where it is processed, releasing the cytosolic domain that acts as a transcription factor [11]. ATF6 controls a subset of UPR-targeted genes related to protein folding and quality control mechanisms [28, 29]. Overall, UPR signaling responses integrate information about the nature and intensity of the stress stimuli to modulate the expression of a large spectrum of partially overlapping target genes that orchestrate adaptation to stress or trigger cell death programs [12].

## 4. ER Stress Signaling in sALS

**Figure 1:**
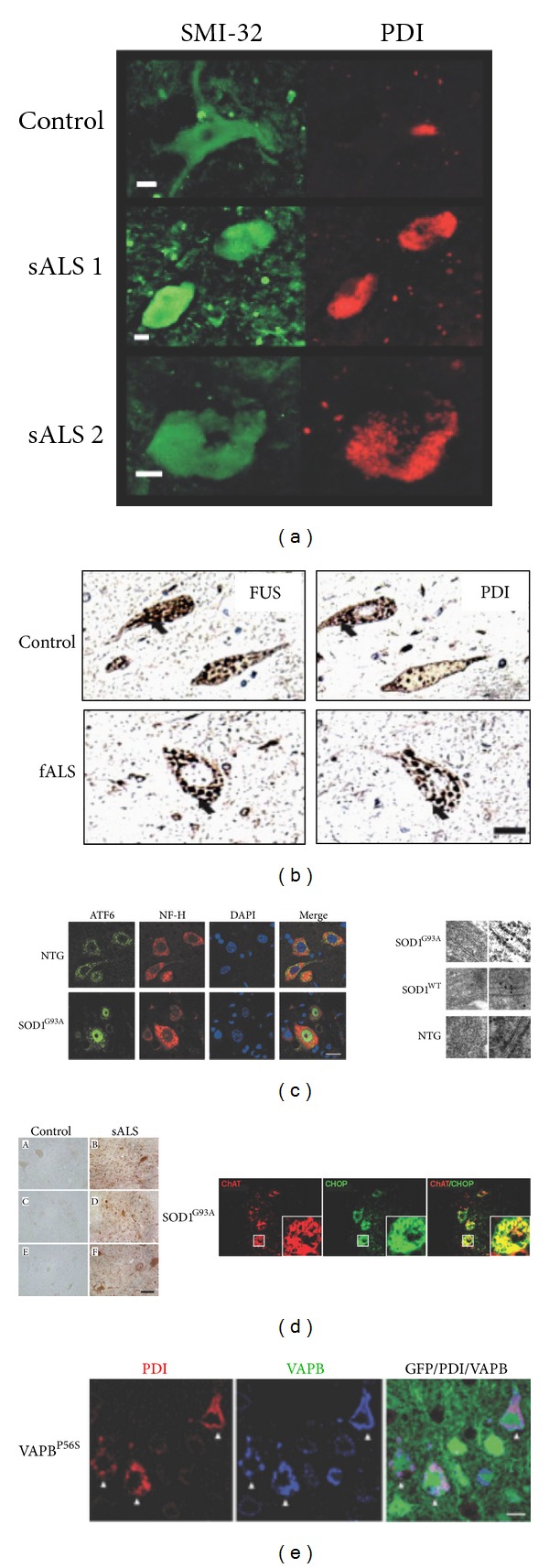
UPR activation in ALS human samples and mouse models of the disease. Several examples of published data indicating the activation of the UPR in SALS human samples and animal models. (a) Immunostaining of spinal cord motoneurons with the neurofilament marker SMI-32 showing PDIA1 (PDI) overexpression in samples from two sporadic ALS patients (sALS) compared with healthy subjects. Scale bar, 10 *μ*m (from Atkin et al. [30]). (b) Immunohistochemistry of spinal cord section from a familial ALS patient (fALS) with a FUS mutation. The colocalization of FUS protein (left panel) and PDIA1 (PDI) protein (right panel) is indicated with black arrows. Scale bar, 40 *μ*m (from Farg et al. [92]). (c) Left panel, immunodetection of the UPR sensor ATF6 (green), neurofilament (NF-H, red), and DAPI (blue) in spinal cord sections from SOD1^G93A^ mutant mice and nontransgenic control animals (NTG). Scale bar, 20 *μ*m. Right panel, SOD1 protein detection in ER lumen by immunoelectron microscopy in SOD1^G93A^ mutant, SOD1 wild-type (SOD1^WT^), and nontransgenic (NTG) mice. Scale bar, 50 nm (from Kikuchi et al. [38]). (d) Left panel, CHOP positive cells detected in spinal cord sections from human sporadic ALS (sALS) patient. Control tissue in (A), (C), and (E). Pictures derived from cervical spinal cord ((A) and (B)), thoracic spinal cord ((C) and (D)), and lumbar spinal cord ((E) and (F)). Scale bars, 65 *μ*m. In the right panel, immunolocalization of CHOP (green) in anti-ChAT (red) positive spinal cord motoneurons from SOD1^G93A^ mutant mice. Scale bar: 40 *μ*m. The areas with a box are shown at higher magnification. Scale bar 10 *μ*m (from Ito et al. [33]). (e) Immunostaining of corticospinal motor neurons from 3-month-old VAPB^P56S^ transgenic mice. Transgene detected with GFP (green), PDIA1 (PDI) (red staining), and VAPB (blue staining). Arrowheads show neurons with accumulation of PDI and VAPB. Scale bar: 20 *μ*m (from Aliaga et al. [50]). Copyright authorization was obtained from each journal for all images.

The involvement of ER stress in sporadic ALS can be inferred from correlative studies in human postmortem tissue. Many reports have identified the upregulation and activation of the three main UPR signaling branches, in addition to the description of elevated levels of ER chaperones and cell death signals linked to ER stress [30–34] (see examples in Figure 1). Ilieva et al. showed enhanced phosphorylation of eIF2*α* and increased levels of the ER foldase PDIA1 along with elevated levels of oxidized proteins in spinal cord of sporadic ALS patients [32]. We also reported the upregulation of the ER foldase ERp57 in sALS and fALS, in addition to the expression of XBP1s and ATF4 [31]. Other groups also described the up-regulation of CHOP in sALS [30, 33] (Figure 1(d)). In line with the aforementioned observations, augmented levels of PERK, ATF6, and IRE1 have been found [30] (Figure 1(c)).

Additional support for the importance of ER stress in ALS pathogenesis comes from ultrastructural studies [35, 36]. Oyanagi et al. detected distended and fragmented ER cisternae in the affected cells of the anterior horn of the spinal cord [35]. In a recent study, Sasaki observed an increased immunostaining for GRP78 (BiP) in affected but also normal-appearing motor neurons from sporadic patients [36]. Strikingly, a detailed examination of ER in normal-appearing motor neurons by electron microscopy revealed dilated ER lumen containing amorphous or granular material [36]. Additionally, ribosome-free membranous structures extending from the ER membrane, electron-dense material resembling Bunina bodies, Hirano bodies, and honeycomb-like structures were observed in patient samples only [36]. Together, these biochemical and morphological evidence correlate the development of ALS with the markers of ER stress. 

## 5. UPR Activation in Experimental Models of ALS

Several laboratories have also shown the occurrence of ER stress in most cellular and animal models of fALS associated with mutations in FUS, TDP-43, SOD1, VAPB, and Ataxin-2 (see examples in [37–50]) (Figure 1). Moreover, in addition to ALS, disturbances in the function of the ER are thought to contribute to cell loss in a number of important human diseases including Parkinson's, Huntington's, and Alzheimer's disease [7, 51]. In this section, we discuss mostly *in vivo* validations of a functional involvement of ER stress in ALS.

In an elegant study from Caroni's group, a systematic transcriptomic analysis was performed using laser dissection of a group of neurons that die early (vulnerable motoneurons) during the course of the disease and a second group that is resistant in a mutant SOD1 model of ALS [52] (Figure 4). This study showed that only affected motoneurons of fALS mouse models were selectively prone to undergo early and chronic ER stress, which was the main molecular signature identified using gene expression profile analysis. Moreover, these changes were detected even before the earliest denervation in asymptomatic animals [52]. In support of this idea, several recent publications suggest that “stressful events” are occurring at the intracellular and intercellular level long before the locomotor defects and the protein aggregation are observed. For example, spinal cord neurons from neonatal SOD1 transgenic mice show hyperexcitability [53, 54], which would be one of the earliest abnormalities found so far.

In addition to UPR markers, Saxena et al. also observed that ALS vulnerable neurons specifically engage stress-management pathways such as protein ubiquitination and hypoxia-related genes, several weeks before this happens in resistant motoneurons [52]. Furthermore, activation of the UPR in vulnerable motoneurons coincides with the activation of microglia [52] (Figure 4(a)). It is unclear whether resistant motoneurons are protected due to differential disease stress inputs (differential degree of stress) or due to particular cellular mechanisms that generate increased resistance to cellular stress. In conclusion, regardless of the cause of motoneuron stress, it is becoming evident that modulation of protein folding stress or the proteostatic capacity of motoneurons may represent a potent therapeutic target to delay the symptomatic phase of ALS. In this context, the use of gene therapy or small molecules to reinforce the stress response capacity is becoming an interesting tool for disease intervention (Figure 4(c)).

**Figure 2:**
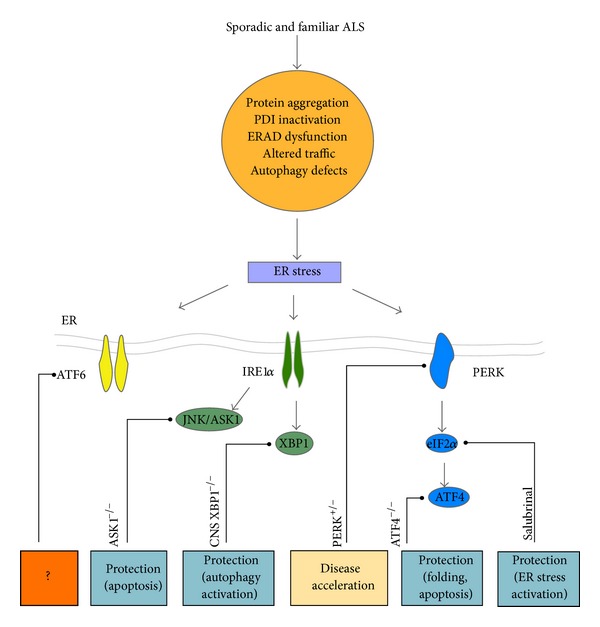
Targeting the UPR in ALS. Different factors may induce ER dysfunction in ALS. For example, abnormal protein aggregation/misfolding, PDIs inactivation by nitrosylation, ERAD dysfunction, altered vesicle traffic, and/or autophagy defects represent conditions that could induce ER stress and lead to an adaptive stress response known as the unfolded protein response (UPR) at early disease stages. The manipulation of different UPR components has revealed a functional contribution of distinct ER stress signaling events in preclinical models of ALS. Genetic targeting of ASK1 (ASK1^−/−^), a downstream signaling component of IRE1*α*, protects against the development of experimental ALS decreasing motor neuron death in the spinal cord of mutant SOD1^G93A^ mice [56]. The deletion in the CNS of the transcription factor XBP1 (CNS XBP1^−/−^) increases the survival of the mutant SOD1^G86R^ mice, associated with reduced accumulation of mutant SOD1 aggregates *in vivo* and enhanced autophagy levels [31]. PERK haploinsufficiency (PERK^+/−^) enhanced the severity of experimental ALS, associated with elevated levels of neuronal loss and mutant SOD1 aggregation [43]. The deletion of the transcription factor ATF4 (ATF4^−/−^) in the SOD1^G86R^ mutant mice delays the appearance of the symptoms and the extended animal survival. These effects were associated to changes in the ER protein folding network and apoptotic genes [63]. In a pharmacological strategy, the treatment of mutant SOD1 mice with a small molecule that selectively induces eIF2*α* phosphorylation, salubrinal, protects against disease progression [52]. No manipulation of ATF6 in animal models of ALS has been described.

In order to understand the contribution of ER stress and the UPR to ALS, many groups have manipulated UPR components and studied the evolution of the disease (Figure 2). The deficiency of the ER stress-related proapoptotic genes *ask1*, *puma*, or *bim* delays ALS in mouse models, possibly by rescuing motoneuron viability [37, 55, 56]. We investigated if deficiency of the transcription factor XBP1 could have an impact on ALS progression by crossbreeding a conditional knockout mouse for XBP1 in the nervous system [57] with transgenic mouse overexpressing mutant SOD1 [31]. Unexpectedly, despite predictions that deletion of this important UPR component would enhance the severity of experimental ALS (i.e., impaired adaptation to ER stress), we observed that the SOD1 mutant offspring that were knockout for XBP1 in the nervous system had delayed disease onset. These effects were associated with reduced accumulation of mutant SOD1 aggregates *in vivo* and in cell culture models and enhanced autophagy levels [31]. In agreement with this concept, we and others have recently reported that the pharmacological activation of autophagy can improve the survival and disease signs of mouse models of ALS, an effect associated with the clearance of abnormal protein aggregates [58, 59]. These findings can be contrasted with the unexpected results obtained from the treatment of the mutant SOD1 mice with another autophagy inductor, rapamycin, in which an accelerated progression of the disease was observed [60]. These results may be explained by the fact that the rapamycin target, mTOR (mammalian target of rapamycin), is involved in diverse cellular processes such as regulation of mRNA translation, cell metabolism, and inflammation, among others [61]. Despite these divergent results, autophagy represents an interesting target for future therapeutic development. 

Other studies have validated a functional contribution of the UPR to ALS with unexpected results (Figure 2). Remarkably, a treatment of mutant SOD1 transgenic mice with salubrinal [52], a small molecule that enhances eIF2*α* phosphorylation [62], led to significant protection against experimental ALS progression [52]. Consistent with this report, *perk* haploinsufficiency (*perk* ^*+/−*^ mice) exacerbated the severity of experimental ALS, decreasing life span. This phenotype was associated with exacerbated neuronal loss and enhanced mutant SOD1 aggregation [43]. In this study, however, the loss of one *perk* allele did not decrease the induction of ATF4 at the early symptomatic stage and only partially reduced ATF4 levels at the end stage of the disease [43]. In agreement with this observation the levels of ATF-4 target genes, such *chop* and *bip* were not altered in PERK^+/−^/SOD1^mutant^ mice [43]. These studies suggest that the effects attributed to *perk* haploinsufficiency in ALS pathogenesis are mostly related to the inhibition of protein translation through eIF2*α* phosphorylation and not due to ATF4 induction.

We also have recently reported the impact of targeting the transcription factor ATF4 in ALS *in vivo* using a full knockout model. Unexpectedly, ATF4 deficiency reduced the probability of the birth of mutant SOD1 mice, suggesting that the UPR may even contribute to mitigating pathological stress during development in this model [63]. On the other hand, the ATF4 knockout/mutant SOD1 transgenic mice that were born showed delayed disease onset and prolonged life span [63]. Consistent with the role of ATF4 in apoptosis, its deficiency completely ablated the induction of BIM and CHOP in mutant SOD1 mice, in addition to induced quantitative changes in the protein homeostasis network. Conversely, ATF4 deficiency enhanced mutant SOD1 misfolding at the end stage of the disease. Thus, PERK signaling may have differential and contrasting effects on ALS pathogenesis, in which eIF2*α* phosphorylation affords protection whereas ATF4 induction may trigger motoneuron apoptosis.

Although the activation of UPR has not been entirely described in animal models expressing TDP-43 mutant proteins [64], in a recent study, the use of drugs to alleviate ER stress showed significant protection against the neurotoxicity induced by mutant TDP-43 in worm and zebrafish models of ALS [65]. The treatment of these animal models with salubrinal or guanabenz, two drugs that sustain eIF2*α* phosphorylation by different mechanisms [62, 66], reduced toxicity and improved motility of worms and fishes expressing mutant TDP-43 [65]. These results, together with those obtained from pharmacological intervention of eIF2*α* in mutant SOD1 mouse models, support the idea that ER stress is a main event in ALS. In summary, these studies illustrate the complex nature of UPR signaling in ALS, clearly demonstrating that targeting specific components of the pathway may have distinct consequences on disease progression [12]. These studies have identified some of the components of the UPR as a potential target to treat ALS.

## 6. A Role of the Glia and Oligodendrocyte UPR in ALS?

The extracellular environment can influence motoneuron fate in the context of ALS as depicted by the interplay between motoneurons and the glia. For example, it is possible to induce ALS pathology in mice overexpressing mutant SOD1 in nonneuronal cells [67]. In cellular assays, supernatant derived from astrocytes/motoneuron cocultures of mutant SOD1 transgenic mice can trigger neuronal death of wild-type neuronal cultures. The toxic factors released from mutant SOD1 primary cells are able to induce hyperexcitability and subsequent cell death [68]. 

Several studies have shown that the expression of mutant SOD1 in astrocytes or microglia regulates the progression of ALS (see examples in [69–71]). A recent study showed that UPR activation also takes place in these glial cells [72]. ER stress markers can be observed particularly in microglia even at early stages of the disease. These results support the idea that UPR may have a broad impact on noncell autonomous aspects of ALS [72].

Recent reports suggest that oligodendrocytes may also play a relevant role in ALS. Extensive degeneration was reported in the gray matter oligodendrocytes in the spinal cord of mutant SOD1 mice prior to the appearance of disease signs [73]. Similar results were observed in ALS human post- mortem tissue [74]. Although new oligodendrocytes were formed, they did not mature and were unable to mediate remyelination. Of note, great advances have been obtained in understanding the role of ER stress in oligodendrocytes in models of multiple sclerosis, where inflammatory reactions trigger demyelination and motoneuron degeneration [75, 76]. IFN-*γ*-dependent activation of the PERK pathway in oligodendrocytes was protective in a mouse model of multiple sclerosis [77]. Moreover, salubrinal also protected against disease progression in the same model [78]. A recent paper confirmed the protective role of PERK pathway against cytotoxic events using a temporally controlled activation of PERK in oligodendrocytes of an experimental model of multiple sclerosis [79]. Similarly, we have recently reported a reduced locomotor recovery in ATF4 or XBP1 knockout models after a spinal cord injury. In addition, gene therapy to deliver active XBP1 into the spinal cord had a significant impact on motor recovery after spinal cord injury which was associated with enhanced oligodendrocyte survival [80]. This is an important finding considering the close relationship of glia and neurons and a possible coordinated/associated stress response between both cell types. These results support the notion that modulating the UPR in a non-cell autonomous manner may also represent an interesting strategy to attenuate ALS progression. This idea remains to be tested. 

## 7. The PDI Family of Proteins and ALS

At the early stages of the UPR activation, the folding capacity of the ER is increased through the up-regulation of the ER chaperons such as BiP/Grp78, Grp94, calreticulin (CRT), calnexin (CNX), and several members of the protein disulfide isomerase (PDI) family [81]. These events reduce ER stress levels by enhancing the folding capacity of the ER or by removing terminally misfolded proteins through ER-associated degradation (ERAD) [82]. In the last years, the role of ER resident chaperons and foldases, in particular some members of the PDI family, has gained an important place in the ALS field. Here we discuss most relevant data revealing a participation of these proteins in the ALS.

A recent genetic screening revealed associations of PDIA1 intronic variants as a risk factor to develop ALS [83]. However, no mechanistic studies were provided to determine the possible impact of these genetic alterations on the disease. PDIs are a large protein family comprised of 21 known members of the thioredoxin superfamily, classified based on sequence and structural homology (reviewed in [84]). Most PDIs have a foldase function and catalyzed disulfide bond formation and, as we will discuss later, can also inhibit protein aggregation and modulate cell viability. Of note, several PDI family members have been involved in neurodegenerative disease such as Parkinson's disease, Alzheimer's Disease, prion-related disorders, and Huntington's disease (review in [85]). Importantly, a proteomic analysis of spinal cord tissue of mutant SOD1 mice reporting PDIA1 and ERp57 (also known as Grp58 or PDIA3) as major up-regulated proteins was the first study suggesting a possible participation of PDIs in ALS [86]. These results were later confirmed by independent study [87]. 

Mutant SOD1 has been shown to accumulate in the ER *in vivo* [38, 86]. In addition, the translocation of SOD1 to microsomal fractions has been reconstituted *in vitro* with purified components [88]. Mutant SOD1 is also secreted to the extracellular space through a classical Golgi-dependent mechanism [41]. Atkin et al. reported a physical interaction between the wild-type and mutant SOD1 and PDIA1* in vivo* [86]. They also showed a colocalization of PDIA1 with mutant SOD1 inclusions. This was also observed in spinal cord samples from ALS patients [32, 89]. Similarly, mutant SOD1 was shown to interact with the ER chaperone BiP in the spinal cord of mutant SOD1 transgenic mice [38]. At the functional level, PDIA1 overexpression in cell culture reduced mutant SOD1 aggregation, ER stress, and also induced cell death [90]. In contrast, the inhibition of PDI with the antibiotic bacitracin [91] increased mutant SOD1 inclusions [86], suggesting that PDIA1 prevents the formation of SOD1 aggregates. Similarly, TDP-43 positive inclusions have been shown to colocalize with PDAI1 in sALS samples [89]. ALS-linked FUS mutant has been also shown to induce ER stress, colocalizing with PDIA1 in cell culture and spinal cord tissue from sALS and fALS cases, in addition to animal models of the disease [92]. Moreover, a physical association between mutant FUS and PDIA1 was reported [92]. It is still unknown if the manipulation of PDI levels will a_ect the progression of experimental ALS *in vivo*.

Modification and inactivation of PDIA1 were also reported in spinal cord tissue from sALS and mouse models of the disease [90]. Similar observations were also described before in brain tissue derived from Parkinson's and Alzheimer's disease patients [93]. It was proposed through cell culture studies that PDI nitrosylation may contribute to the disease by inhibiting the protective roles attributed to these foldases. This abnormal modification of PDI could result from altered nitric oxide synthase activity found in mouse models of the disease [94]. Although PDIs are thought to have a neuroprotective activity, one report suggested that PDIA1 and ERp57 may actually have a pro-apoptotic activity in models of Alzheimer and Huntington's disease [95]. Accordingly, UPR activation in microglia correlated with an increase of PDIA1 protein and neurotoxicity [72]. These data suggest that future therapeutic manipulation of the UPR should examine in more detail its impact on glial cells.

**Figure 3:**
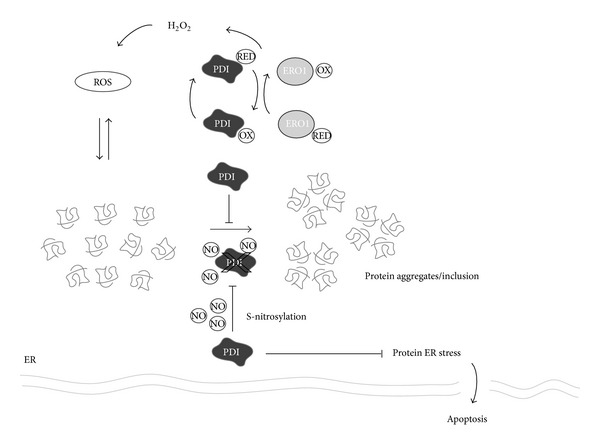
Possible role of protein disulfide isomerases (PDIs) in ALS. PDIs are up-regulated in diverse models of ALS in addition to tissue from patients. They also have been suggested as possible biomarkers to monitor disease progression using body fluids. PDIA1, in the figure PDI, colocalized with protein inclusions containing FUS, TDP43 or SOD1 in human tissue and mouse models of ALS. The exact contribution of PDIs to ALS is currently a matter of debate. PDIs could have a protective role through decreasing protein aggregation and global ER stress. S-nitrosylation appears to inactivate PDI and contribute to ER stress. Reactive oxygen species (ROS) produced from redox folding (disulfide bond formation) may also contribute to the generation of protein aggregates.

The formation of disulfide bonds by PDIs inside the ER requires specific redox conditions and fine balance between the oxidized and reduced states of PDIs [96–100]. The ER is an extremely oxidizing environment compared with the cytoplasm, and the maintenance of its redox state relies on PDI activity of the formation of the disulfide bonds. The generation of disulfide bonds is highly regulated and involves the enzyme ERO1, which is an important oxidase for disulfide formation [101]. The perturbation of the redox status of the ER is deleterious for the proper cell function and there are tight mechanisms to buffer the possible redox fluctuations [102]. We have recently described that ATF4 deficiency alters the redox status of the cell and also the ER as measured by monitoring H_2_O_2_ levels, a subproduct of the PDI/ERO1 cycle [63]. Of note, the treatment of motoneuron cells with the antioxidant trolox is able to revert the enhanced aggregation of mutant SOD1 observed after knocking down ATF4. In addition, overexpression of ERO1 also modulated mutant SOD1 aggregation [63], suggesting that the manipulation of ER redox state can impact the misfolding of mutant SOD1. Taken together, these data suggest that PDIs may play a significant role in ALS by affecting different aspects of cell physiology including protein aggregation, cell survival, and the redox status of the ER (Figure 3).

## 8. ER Stress Signaling in sALS: Novel Biomarker for Disease Prognosis?

Early studies have shown that several ER chaperones can be secreted to the extracellular space upon stress [103]. Recently, PDIA1 levels have been reported to be up-regulated in the cerebrospinal fluid (CSF) of ALS patients [30]. Interestingly, Vijayalakshmi et al. showed the induction of ER stress in spinal motor neurons exposed to CSF of sporadic ALS patients [34]. This fact suggests that measuring stress factors in CSF may represent an interesting tool to monitor ALS disease progression. There is a current need for biomarkers of ALS to assess, on a quantitative manner, disease prognosis and the efficacy of clinical trials.

In a recent proteomic screening searching for biomarkers in blood samples from sALS patients, the up- regulation of the ER stress-responsive chaperones PDIA1, ERp57, and other chaperones was observed [104]. Similar changes were also seen in mononuclear cells from blood of mutant SOD1 mice. It was demonstrated that TDP-43, cyclophilin A, and ERp57 are strongly associated with disease course in a longitudinal study in ALS patients and control subjects, ERp57 having the best score [104]. These two studies open the interesting possibility of monitoring stress signatures to diagnose and monitor progression of ALS. 

## 9. Perspective

**Figure 4:**
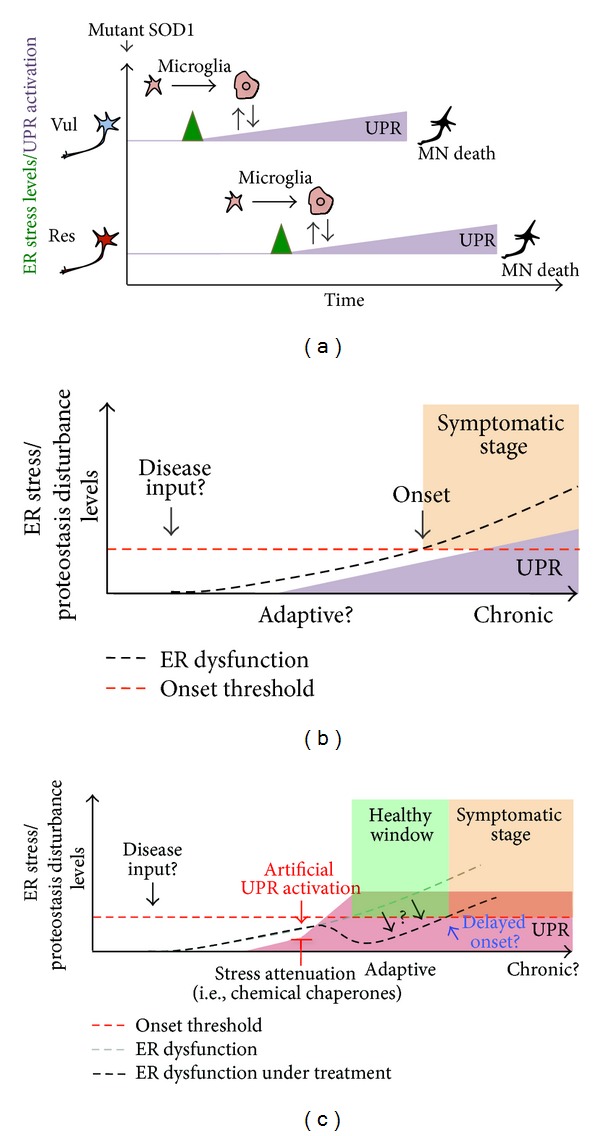
ER stress and the selective neuronal vulnerability in ALS. (a) Schematic representation of ER stress levels (green label) and UPR activation (purple label) in the two subgroups of neurons that have been identified in mutant SOD1 mouse models of ALS: one population that dies early (vul, vulnerable, blue) and another that dies later during disease progression (res, resistant, red). Activation of stress markers is a common feature detected in Vul and Res motoneurons. However, vul motoneurons express these stress markers earlier than de res neurons. The UPR is activated in both subgroups of neurons. UPR activation also correlates with microglial activation in both groups. It is not known what determines the resistance of Res cells in the disease. (b) Time-course of ER stress levels and UPR activation in familial ALS models. ER stress and protein disturbance increase during ALS progression (“ER dysfunction,” black dashed line). During the presymptomatic stage of the disease, UPR activation might represent an adaptive response that attenuates ER stress levels. Over time, the stress condition exceeds the capacity of the cell to manage protein folding stress and pro-apoptotic pathways are activated. This shift “onset threshold” in UPR signaling regulation could be associated with motoneuron dysfunction/loss and the onset of the disease. During the symptomatic stage, a strong and chronic UPR activation occurs. (c) Possible therapeutic approaches to modulate the UPR in ALS. An earlystage preventive treatment may modulate UPR levels to enhance the adaptive capacity of motoneurons and reduce ER stress levels or other proteostasis disturbances. This may delay disease onset and disease evolution “healthy window”. The therapeutic approaches include gene therapy to deliver active UPR components and the use of smallmolecules that selectively activate specific UPR signaling branches (pharmacologic approaches) or act as chemical chaperones to alleviate global ER stress. This reduction in ER stress levels in motoneurons could also be achieved by modulating glial UPR.

ER dysfunction is currently viewed as a relevant factor driving diverse diseases of the nervous system, representing an important niche for drug discovery. Due to the fact that the type, intensity, and temporality of ER stress stimuli determine how the UPR integrates information towards controlling cell fate, this pathway offers interesting targets to modulate both cell survival and death mechanisms. Depending on the disease context, targeting strategies may involve attenuation of ER stress levels, inactivation of pro-apoptotic components of the UPR, or the enhancement of UPR signaling responses toward adaptation to stress (Figure 4). The scenario in ALS is very complex. Genetic and pharmacological manipulation of the pathway in preclinical models of the disease supports the idea that the UPR may contribute to both cell viability of stressed cells and also the elimination of motoneurons when there is irreversible damage. More research is needed to understand the consequence of manipulating the UPR to validate the pathway as a target. For such step, it is essential to define the optimal targets to alleviate ER stress in ALS. Importantly, it is becoming clear that sporadic and familial ALS, regardless of the specific genetic alteration, may converge into alterations on ER function, offering unique therapeutic opportunities. The fact that mutations in PDIA1 gene were recently described in ALS patients suggests a causative role of proteostasis defects at the ER. Supporting this notion, mutations in two important proteins involved in the degradation of misfolded proteins, Ubiquilin1 [105] and p62 [106], have been found in ALS cases. Predicting and defining the possible side effects of manipulating the UPR at the systemic levels remains an important subject for future validation of the pathway as a drug target and move forward into the development of human therapies.
